# Analysis and prediction of immune cell infiltration characteristics in COPD: Folium isatidis and its active ingredients are able to combat lung lesions caused by COPD by correcting immune cell infiltration

**DOI:** 10.3389/fmed.2025.1584411

**Published:** 2025-04-23

**Authors:** Faqin Wang, Weichen Zhang, Yujie Huang, Xianbang Hou, Liwei He, Huiqin Xu

**Affiliations:** ^1^Department of Pharmacology, School of Pharmacy, Nanjing University of Chinese Medicine, Nanjing, China; ^2^Department of Pharmacology, Nanjing University of Chinese Medicine Hanlin College, Taizhou, China

**Keywords:** immune infiltration, pulmonary function, chronic obstructive pulmonary disease, network pharmacology, indirubin, folium isatidis

## Abstract

**Objective:**

As a respiratory disease, chronic obstructive pulmonary disease (COPD) has become a common fatal disease worldwide. We aimed to screen active traditional Chinese medicines (TCMs) for the treatment of COPD by COPD-related transcriptome gene chip analysis and verify their therapeutic activity for COPD.

**Methods:**

We used bioinformatics analysis to predict potential therapeutic TCMs based on the immune cell infiltration characteristics in COPD. Further, BALB/c female mice were divided into different treatment groups to investigate the effect of indirubin (IDR) and FI on COPD. After 12 weeks of intranasal lipopolysaccharide instillation and passive smoking, we started giving drug treatment to mice, including distilled water (control and model groups), dexamethasone, IDR and FI. The lung function, visceral index, degree of lung tissue damage, and immune cell infiltration were assessed.

**Results:**

We identified 109 differential genes, 22 immune cells, and 50 TCMs associated with the alleviation of COPD. The *in vivo* experimental results showed that IDR and FI had protective effects against lung injury in mice and could inhibit COPD. The mechanism of this effect may be related to their ability to regulate the proportion and distribution of immune infiltration of B lymphocytes, CD4+ and CD8+ T lymphocytes, Treg cells, NK cells, myeloid-derived suppressor cells, and eosinophils.

**Conclusion:**

IDR and FI can ameliorate disease development in COPD model mice by regulating immune cell infiltration. This offers an experimental groundwork for delving deeper into the mechanisms through which TCMs impact COPD treatment and for identifying possible therapeutic drugs for COPD.

## 1 Introduction

Chronic obstructive pulmonary disease (COPD) represents a complex and multifactorial respiratory disorder characterized by progressive airflow limitation and dyspnea. Due to its significant association with elevated morbidity and mortality rates, COPD has emerged as the third most prevalent cause of global mortality (1). Exposure to environmental particles, particularly cigarette smoke, is the leading cause of COPD. These patients exhibited progressive airflow obstruction due to small airway fibrosis (emphysema), alveolar wall destruction, and chronic inflammation (2).

The leaves of *Isatis indigotica* Fortune (Brassicaceae) are listed as Folium isatidis (FI) in the Chinese Pharmacopoeia. FI have been used extensively in traditional Chinese medicine (TCM) for a long time. FI has a bitter taste; is cold in nature; and belongs to the liver, heart, stomach, and spleen meridians. Its TCM properties are primarily being relied upon in clinical settings to treat immune system diseases, such as lupus erythematosus and psoriasis, respiratory tract infections, and acute infectious hepatitis. FI also has detoxification and heat elimination properties and can cool the blood and eliminate some spots on the body caused by heat (3, 4). Indirubin (IDR) is a well-known major active component present in FI, and it has already been demonstrated to have some anti-inflammatory effects. This activity comes from IDR inhibition of some inflammatory pathways, thereby reducing the expression of inflammatory factors, and this effect has also been demonstrated in certain respiratory diseases (5). In addition, IDR also has some activity in immunosuppression and anti-tumor.

The deconvolution method was employed in the present investigation to quantitatively assess and systematically examine the gene expression profiles in individuals diagnosed with COPD and obtained the immune cell infiltration characteristics to predict TCM drugs and verify their activities. Network and *in vivo* pharmacology experiments were used to elucidate the mechanism of COPD and the efficacy of FI, and its major active component IDR, which lay the groundwork for the advancement and implementation of alike therapeutic approaches.

## 2 Materials and methods

We first used the Gene Expression Omnibus comprehensive gene expression database (GEO, https://www.ncbi.nlm.nih.gov/geo/) with the keyword “COPD” for retrieval and selected the appropriate gene chips for research. Sample gene expression matrix was used to identify key differential genes, and functional enrichment analyses, including Gene Ontology (GO) and Kyoto Encyclopedia of Genes and Genomes (KEGG) pathway analyses, were systematically performed to elucidate immune-related biological processes and associated signaling pathways. Simultaneously, we obtained the characteristics of immune cell infiltration by calculation and analysis with gene expression profiling. According to these analyses, we screened for TCM drugs with possible efficacy against COPD. Finally, we performed flow cytometry to verify and investigate the regulatory effect of related TCM drugs on immunity against COPD.

### 2.1 Reagents

Lipopolysaccharide (LPS, IL2020) was purchased from Solarbio (Beijing, China). Cigarettes were purchased from Hongyunhonghe Tobacco Co., Ltd. (Yunnan, China). Sodium carboxymethyl cellulose (20200713), ethanol anhydrous (100092683), xylene (10023418), and neutral gum (10004160) were purchased from Sinopharm Chemical Reagent Co., Ltd. (Beijing, China). Hematoxylin and eosin (H&E) staining solution (G1005), differentiation fluid (G1005-3), and reversed blue solution (G1005-4) were purchased from Yiang Biotechnology (Shanghai, China). The Anti-Mouse antibodies CD3-APC (E-AB-F1013E), CD4-FITC (E-AB-F1097C), CD8a-PC5.5 (E-AB-F1104I), CD25-PE (E-AB-F1102D), Foxp3-APC (E-AB-F1238E), CD11b-FITC (E-AB-F1081C), Ly-6G/Ly-6C-APC (E-AB-F1120E), CD19-PE (E-AB-F0986D), NK1.1-PE (E-AB-F0987D) and F4/80-APC (E-AB-F0995E) were purchased from Elabscience (Wuhan, China).

FI (200901) were purchased from the Tongling Market in Anhui and certified by Professor Chen Jianwei of Nanjing University of Chinese Medicine. The quality standards were in accordance with the provisions of Chinese Pharmacopoeia. IDR (S30589) and Dexamethasone (S17003) was purchased from Yuanye (Shanghai, China).

### 2.2 Equipment

An optical microscope (CKX41-A32PH) was purchased from Olympus (Tokyo, Japan). A tabletop centrifuge (TG1850-WS) and electronic balance (YP B6002) were purchased from Shanghai Guangzheng Medical Equipment Co., Ltd. (Shanghai, China). Other equipment used included a small animal laryngoscope (Shanghai Yuyan Instruments Co., Ltd.), flow cytometer (Beckman Coulter, Brea, CA, United States), and high-speed centrifuge (Thermo Fisher Scientific, United States). Smoking exposure cabinets (50 × 40 × 40 cm) were self-made.

### 2.3 FI extraction

To obtain the total extract of FI, 1,000 g FI powder was soaked in thrice its volume of 2.5% ammonia solution for 12 h and was extracted thrice with an 8-fold volume of methylene chloride for 1 h each time. The resulting sample was dried in a vacuum drying oven to obtain the FI extract, with a yield of 24.1 g (2.41%).

### 2.4 Animal modeling and grouping

We obtained SPF female BALB/c mice weighing 19 ± 2 g [certificate of Conformance No.: SCXK (Lu) 20160001] from Qinglongshan Animal Breeding Farm (Jiangning District, Nanjing, China). Mice were fed for 7 days with free access to eating food and drinking water and housed at a temperature of 23 ± 2°C and a humidity of 55–65% in well-ventilated cages that were cleaned regularly.

After adaptive feeding for 1 week, 70 mice were randomly divided into the Control (CON), Model (MOD), Dexamethasone (DEX), Indirubin low-dose (IDR-L), Indirubin high-dose (IDR-H), FI low-dose (FI-L), and FI high-dose groups (FI-H), with 10 mice in each group.

COPD was induced in all groups of mice except CON after 12 weeks of modeling: 2 mg/kg LPS was intranasally instilled into mice in each group at the same time of week 2, week 4 and week 6. From week 1, mice in each group were placed in a smoking exposure cabinet for cigarette smoking for 40 min, eight cigarettes/time (tar 14 mg/cigarette, nicotine 1.1 mg/cigarette), twice a day. CON mice were intranasally instilled with distilled water at the same time and maintained in a smoke-free normal environment.

From week 7 of passive smoking, we started giving drug treatment to mice. The daily doses of raw FI administered were 9–15 g. The maximum dose of 15 g was taken, and the dry extract yields for FI was 2.41%. According to the “Conversion Table of Drug Doses between Humans and Animals,” the dosage for mice equivalent to the conventional clinical dosage for adults was calculated. The equivalent dose was used for the low-dose group, and four times this dose was administered to the high-dose group. The low dose of FI was 46.60 mg/kg/day, and the high dose of FI was 186.40 mg/kg/day, and the low dose of IDR was 10 mg/kg/day, and the high dose of IDR was 40 mg/kg/day, which were administered by gavage. The DEX group was intraperitoneally injected with 1.5 mg/kg/day dexamethasone, and the CON and MOD groups were administered by gavage with an equal volume of distilled water.

### 2.5 Analysis of immune infiltration of COPD

#### 2.5.1 Acquisition of therapeutic differential genes for COPD

First, we searched the GEO comprehensive gene expression database with “COPD” as the keyword, selected the GSE212331 gene chip as the object of this study, and converted probes into gene names using the GPL10558 platform. The sample grouping of the gene chip included the COPD group and a blank control group. Then, a *t*-test and variance analysis were conducted based on the gene probes, with a screening criterion of |log_2_FC| >1 and corrected *p* < 0.05. The genes were categorized into upregulated and downregulated groups based on their positive and negative log_2_FC values. Upregulated and downregulated genes were visualized using heat maps and volcano plots.

#### 2.5.2 Construction of the protein interaction network

We analyzed the correlation between the obtained differential genes in the STRING database,^1^ analyzed the results with Cytoscape-3.10.0 software, and calculated the degree value (DC) of each target gene using the CentiScaPe plug-in. The top 20 genes were selected as core differential genes, and protein interaction networks for core differential genes were constructed using Cytoscape software for visualization.

#### 2.5.3 Enrichment analysis of core genes

Using R-4.1.3, the “clusterProfiler” packet was retrieved, and GO and KEGG analysis of differential genes was conducted in R.

#### 2.5.4 Characteristics of immune infiltration in COPD

We used the CIBERSORT deconvolution method to simulate the gene expression profiles of COPD 1,000 times and obtained the transcription characteristic matrices of infiltration of 22 immune cells. The Kruskal–Wallis rank sum test was used to assess the Pearson correlation coefficient between samples with credible data (*p* < 0.05). Differences between the COPD group and the control group were analyzed (6).

#### 2.5.5 Prediction of traditional Chinese medicines

We imported data of the core differential genes and GO-enriched immune-related biological processes of samples from COPD patients and paired healthy patients into the Coremin Medical database^2^ and analyzed and screened TCMs with possible mechanisms of immune intervention. Then, we counted the meridian affinity distribution of the screened TCMs to facilitate analysis of the screened TCMs according to the syndrome.

### 2.6 General signs, body weight, lung function assessment and organ index of animals

General signs such as moving hair and secretions were observed twice a day after the initiation of drug administration. At the end of drug administration, lung function was measured using an animal lung function testing system (PFT-M, TOW-INT, Shanghai, China). We connected mouse anesthetized and tracheotomized to a detection system and observed and detected their resistance of inspiration (RI), dynamic lung compliance (Cdyn), inspiratory capacity (IC), forced vital capacity (FVC), peak expiratory flow (PEF), and FEV0.2/FVC. After sacrificing the mice, their lungs, thymus, and spleen were measured, and the organ index was calculated using the formula: organ index = [organ weight (mg)/mouse body weight (g)] × 100%.

### 2.7 Morphological observation of animal lung tissue

The weighed lung tissue was fixed in 4% paraformaldehyde for 1 day, removed and cut into small pieces of tissue, dehydrated and hyalinized using conventional gradient ethanol, embedded in paraffin and sectioned, placed on glass slides, dried and dewaxed, cleaned with gradient ethanol, and stained with H&E solution. The histopathological changes were then assessed under a light microscope.

### 2.8 Flow cytometry

At the end of drug administration, the right lungs of each group were excised and immersed in 1 mL of 1 mg/mL collagenase type I solution. The lungs were cut into pieces and shaken for 2 h (37°C, 60 × g). After filtration with a 300-mesh nylon mesh, we obtained a single-cell suspension of mouse lung tissue. We then added 3 mL of red blood cell lysate, placed it at 4°C for 5 min, centrifuged at 800 × g (Thermo Fisher MicroCL 21R) for 10 min to remove red blood cells, and adjusted the cell concentration to 1 × 10^7^ cells/mL. Then 100 μL cell suspension was taken from each sample tube, and 20 μL each of CD3-APC and CD4-FITC, CD3-APC and CD8a-PC5.5, CD25-PE and Foxp3-APC, CD11b-FITC and Ly-6G/Ly-6C-APC, CD3-APC and CD19-PE, CD3-APC and NK1.1-PE and CD11b-FITC and F4/80-APC were added for cell membrane surface labeling. These cell membrane surface markers were used to identify CD4^+^ T lymphocytes, CD8^+^ T lymphocytes, regulatory T (Treg) cells, myeloid-derived suppressor cells (MDSCs), B lymphocytes, natural killer (NK) cells and eosinophils (Eos), respectively. The cells were maintained on ice in darkness for a duration of 30 min to allow for incubation. After fixation at ambient temperature (10–30°C) for 30 min, staining was performed, and the membrane was broken. Cells were incubated with staining buffer for 30 min, washed twice with buffer, and finally analyzed through flow cytometry by scanning 10,000 cells and using the default settings as conditions for cell labeling and analysis.

### 2.9 Statistical methods

Statistical analysis was conducted using the Statistical Package for the Social Sciences (SPSS) (version 25.0). Data were expressed as “mean ± SD.” Normality and homogeneity of variance were tested, and a two-tailed Student’s *t*-test was used for pairwise comparisons, while a one-way ANOVA was employed for multiple comparisons. *p* < 0.05 were considered statistically significant.

## 3 Results

### 3.1 Differential gene acquisition and protein interaction network construction in patients with COPD

**Figure 1:**
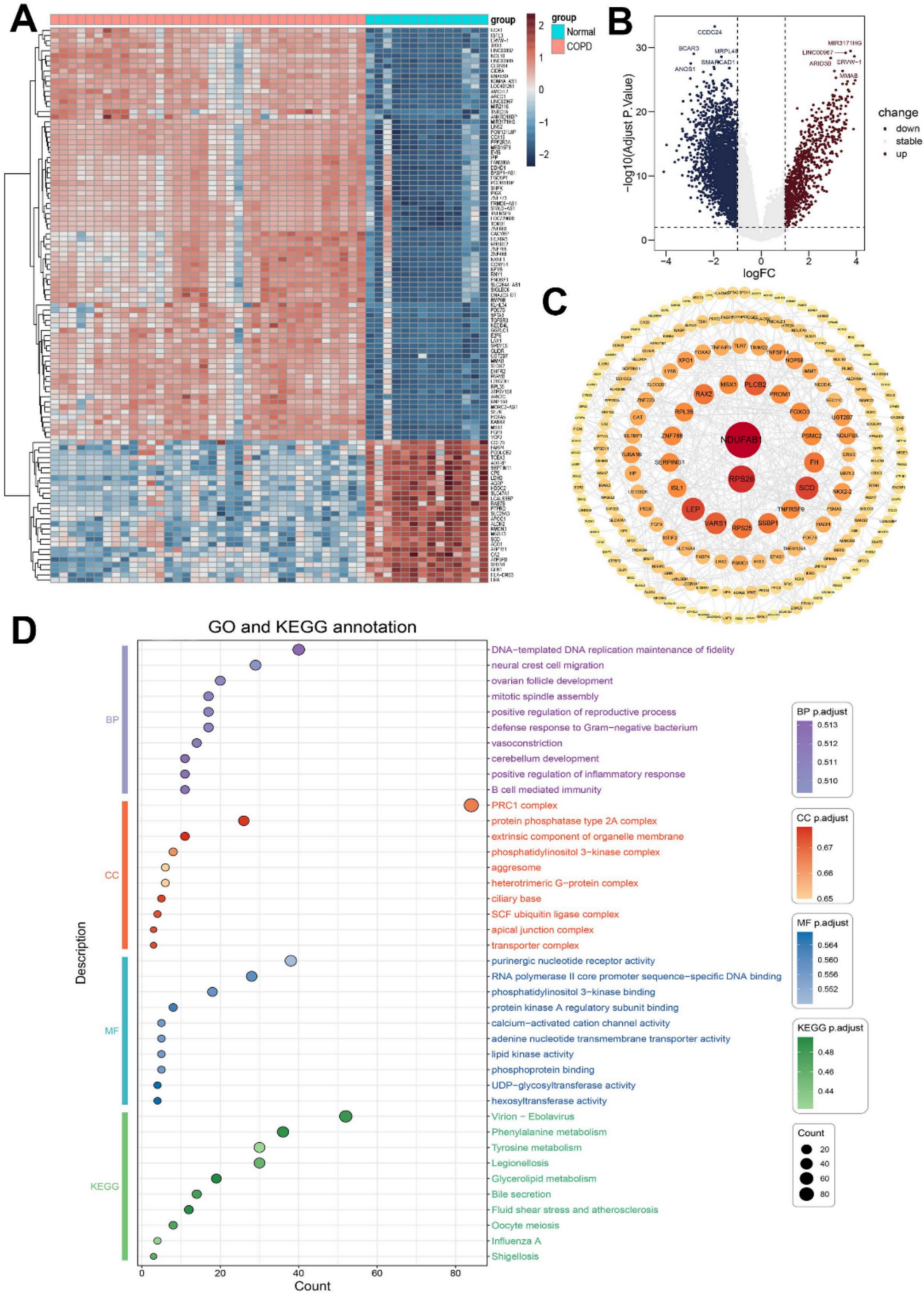
Differentially expressed gene analysis in COPD patients. **(A)** Heat map of differentially expressed genes. **(B)** Volcano plot of differentially expressed genes. **(C)** Protein network interaction analysis of differentially expressed genes. **(D)** GO and KEGG analysis of differentially expressed genes.

Following our screening procedure, a total of 293 genes were identified as differentially expressed between COPD group and the control group. We further limited the screening conditions and selected the differentially expressed genes of TOP109 as significantly differentially expressed genes (Figures 1A,B).

The STRING database was used to construct a protein–protein interaction (PPI) network analysis for significantly differentially expressed genes. The top 10 genes selected as core target genes were NDUFAB1, RPS26, LEP, SCD, PLCB2, RAX2, SSBP1, FH, TNFRSF9, VARS1 (Figure 1C).

To investigate the underlying biological mechanisms and elucidate the associated signaling pathways enrichment of differentially expressed genes in COPD, we conducted GO and KEGG enrichment analyses. The top 10 enriched terms in each category, including molecular function (MF), biological process (BP), and cellular component (CC), were selected for subsequent visualization and analysis (Figure 1D). Through GO enrichment analysis, we observed that the biological processes of differentially expressed genes were closely associated with immune function, including positive regulation of inflammatory response, B cell-mediated immunity, and defense response to Gram-negative bacteria. In the KEGG enrichment analysis results, various pathogenic microorganism-related diseases were found to be linked to these differentially expressed genes.

### 3.2 Analysis of immune infiltration characteristics of COPD

**Figure 2:**
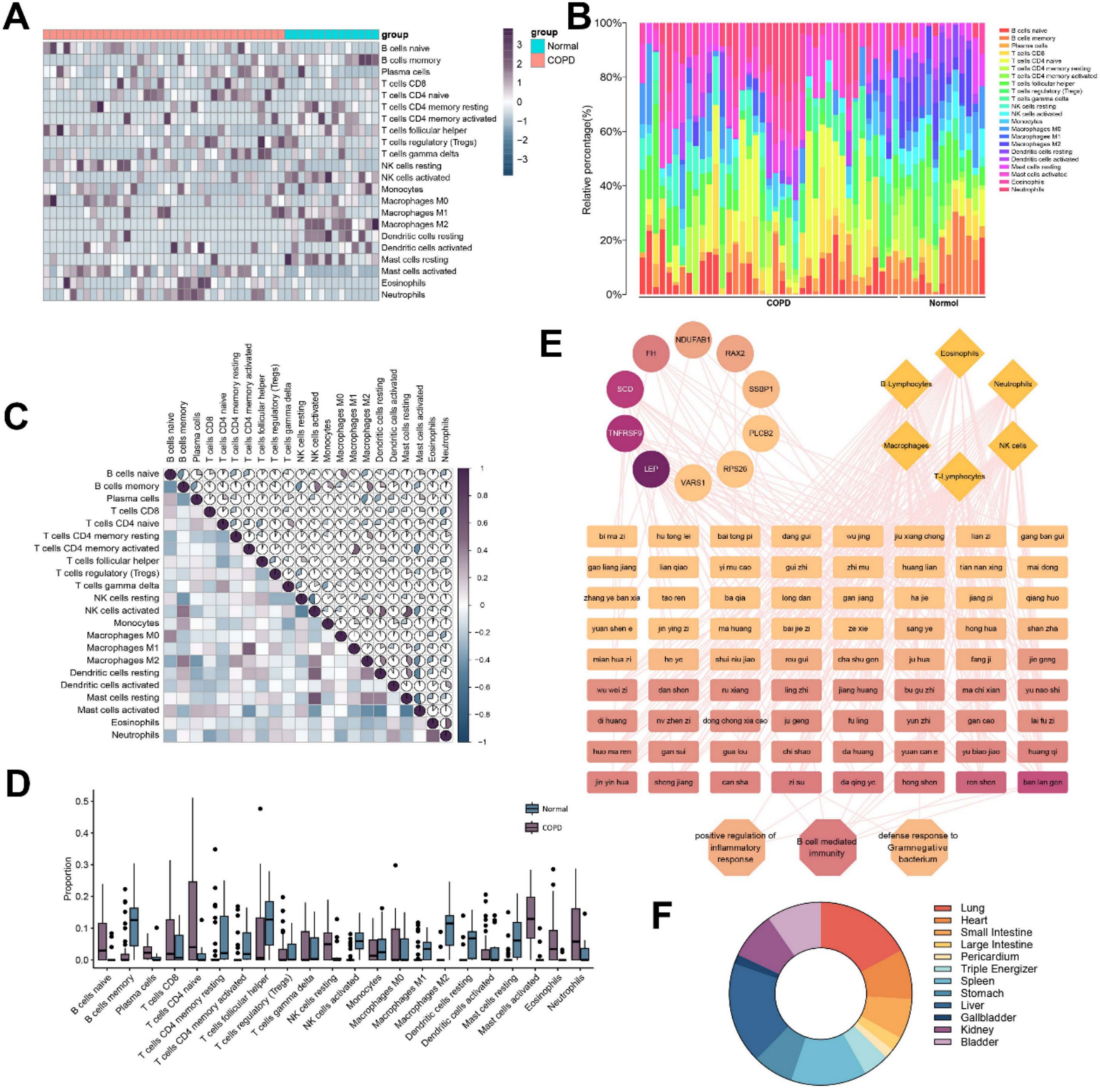
Analysis of immune cell infiltration in patients with COPD and prediction of potential Chinese herbs for COPD. **(A)** Heat map of immune cell infiltration calculated based on differentially expressed genes in COPD patients. **(B)** Accumulation plot of immune cell infiltration ratio. **(C)** Heat map of immune cell infiltration correlation. **(D)** Level of immune cell infiltration. **(E)** Potential therapeutic TCMs prediction based on differential genes, biological processes, and immune cells. **(F)** Predicting meridian analysis of TCMs.

We screened 50 reliable samples. As shown in Figures 2A,B, the immune cell proportion box plot further shows the infiltration proportions of 22 immune cells from 50 reliable samples. In the immune cell heat map and the immune cell proportion box plot, the right 14 samples were the Normal group, and the left 36 samples were the COPD group. The results showed that activated CD4^+^ T cells and activated NK cells were decreased and CD8^+^ T cells and naive B cells levels were increased in COPD patients compared to controls. In parallel, activated mast cells, Eos, and neutrophils levels were found to be increased in COPD patients.

Analysis of immune cell infiltration revealed a positive correlation between activated CD4^+^ T memory cells and M1 macrophages, while activated NK cells showed a strong positive correlation with both resting mast cells and resting dendritic cells. On the other hand, NK cells were negatively correlated with B memory cells, and activated mast cells were negatively correlated with both CD4^+^ T memory cells and dendritic cells (Figure 2C).

We compared the differences in immune infiltration between the COPD group and the control group. According to the box plots in Figure 2D, it is more visually shown that plasma cells, CD8^+^ T cells, activated mast cells, Eos, and neutrophils increase to varying degrees, while B memory cells, T helper cells, CD4^+^ T memory cells, activated NK cells, and macrophages decrease significantly when samples from patients with COPD were compared with those from the normal population.

### 3.3 Screening for TCMs based on core differential genes, immune-related biological processes, and signaling pathways

We identified 50 TCMs possibly associated with these core genes, BPs, signaling pathways and immune cells. Subsequently, we classified the screened TCMs according to their meridian affinity for further analysis (Figure 2E). The high degree values of *Isatis indigotica* Fortune (Brassicaceae), *Platycodon grandiflorus* (*Jacq*.) A. DC. (Campanulaceae), *Panax ginseng* C.A.Mey (Araliaceae), FI, *Chrysanthemum morifolium* Ramat. (Compositae) and *Cinnamomum cassia* Presl (Lauraceae) in the network suggest they are closely related to the immune infiltration of COPD, and most of these TCMs show affinity to the liver and lung meridians (Figure 2F).

### 3.4 Effects of IDR and FI on the physical signs, lung function and visceral index in mice with COPD

Qian Yi in the Song Dynasty wrote the “Key to Therapeutics of Children’s Diseases” records: “Chest blockage, shortness of breath, wheezing cough, should first evacuate the lungs, and then diverge wind cold. Use Xiebai San and Daqing Gao to evacuate the lungs. When the lungs are no longer typhoid fever, the chest is no longer blocked.” Therefore, in the treatment of asthma, it is necessary to evacuate the real evil in the lung first, and then evacuate the evil of wind and cold. Dissipate the evil of wind-cold can be used Daqing ointment. The monarch drug in Daqing Gao is FI, so we combined the results of gene chip screening to guess that FI has a certain therapeutic effect on COPD, and FI was subsequently investigated as a potential therapeutic effect in COPD.

**Figure 3:**
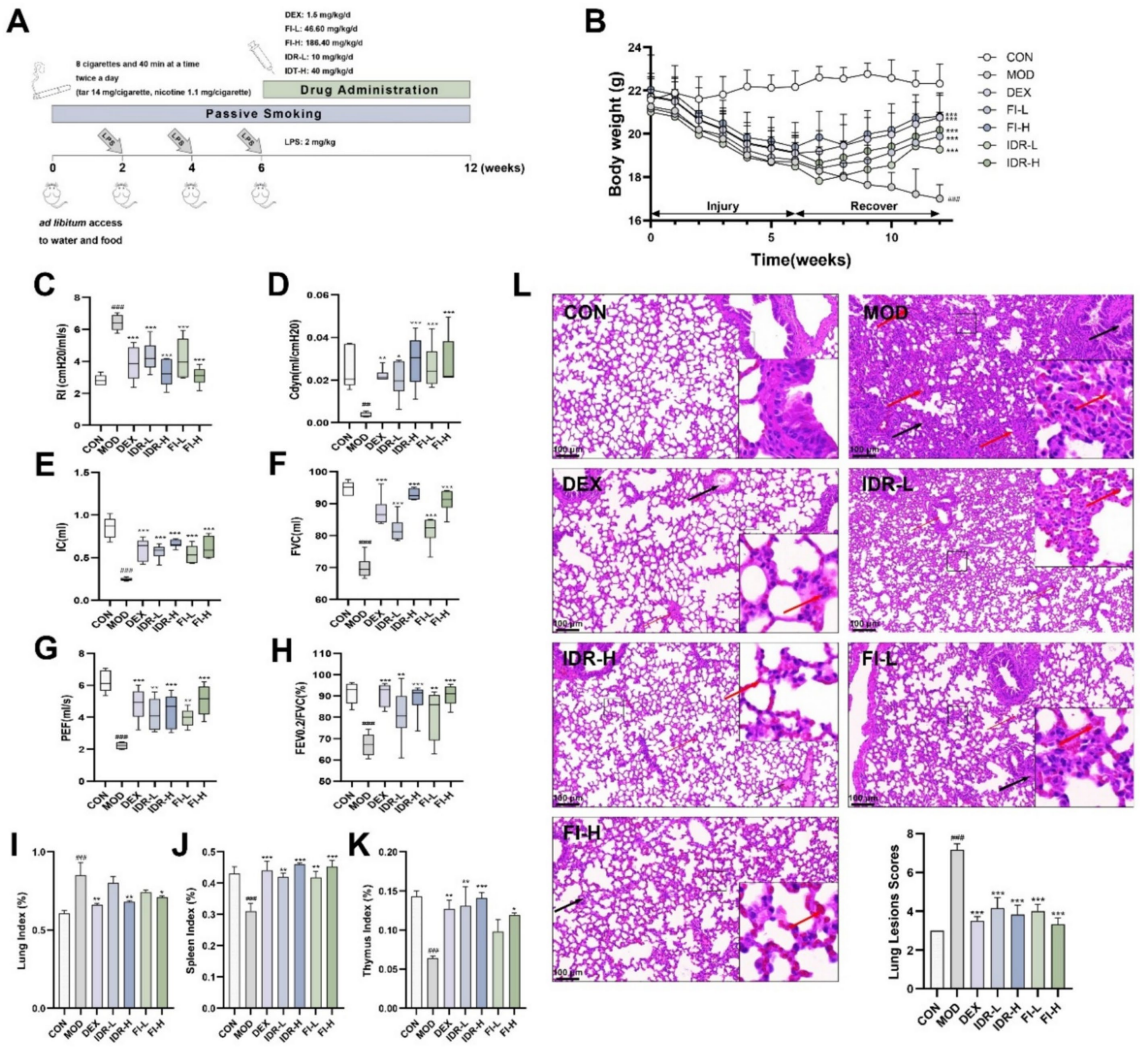
IDR and FI mitigate the development of COPD. **(A)** Modeling and treatment of COPD mice. **(B)** Body weight changes in mice. **(C)** RI, **(D)** Cdyn, **(E)** IC, **(F)** FVC, **(G)** PEF, and **(H)** the ratio of FEV0.2/FVC in mice. Analysis of **(I)** lung index, **(J)** spleen index and **(K)** thymus index in mice. **(L)** H&E staining and lesions scores of mouse lung tissue. Red arrows: inflammatory cell infiltrates. Black arrows: mucus exudation. Data were expressed as mean ± SD, ^##^*p* < 0.01, ^###^*p* < 0.001 vs. CON group, ^*^p < 0.05, ^**^p < 0.01, and ^***^p < 0.001 vs. MOD group (*n* = 6).

In addition to the CON, we developed a mouse model of COPD and administered the corresponding drugs to treat COPD, which are shown in Figure 3A. Compared with CON mice, MOD mice showed a significant and sustained trend of body weight loss under combined LPS and smoke stimulation for 12 weeks (*p* < 0.001), accompanied by physiological state changes such as sparse hair, decreased gloss and decreased activity. In contrast, IDR or FI intervention starting at week 7 significantly reduced this effect, resulting in reversal of body weight in these mice to varying degrees (*p* < 0.001) (Figure 3B). More intuitively, these mice gradually regained luster and behavioral activity.

At the same time, we monitored lung function in mice. As shown in Figures 3C–H, in COPD mice, we found that RI was significantly increased (*p* < 0.001), whereas Cdyn, IC, FVC, PEF, and FEV0.2/FVC were decreased (*p* < 0.01, *p* < 0.001). However, the changes caused by this COPD were relieved and cured to varying degrees after the introduction of IDR and FI (*p* < 0.05), suggesting that both IDR and FI improved the pulmonary function of COPD mice.

Pulmonary edema was evaluated using lung index, while the immune function was evaluated using spleen and thymus indexes. COPD exhibited a significant elevation indices in lung index (*p* < 0.001), accompanied by a significant reduction in spleen and thymus (*p* < 0.001) compared to control mice. Intervention with IDR-H and FI-H reduced the impact of COPD (*p* < 0.05, *p* < 0.01, *p* < 0.001). These findings indicate that IDR and FI reduced pulmonary edema and inflammation in the lungs, prevented spleen and thymus atrophy, and improved immune organ function (Figures 3I–K).

### 3.5 IDR and FI alleviated pathological changes in lung tissues of COPD mice

Figure 3L shows that the alveolar architecture within the lung tissue of mice exhibiting COPD phenotypes, inflammatory cell infiltration is visible in the interstitium, and the alveolar wall is thickened. Following treatment with IDR and FI, the damage to the alveolar structure was alleviated, along with the congestion and alveolar wall thickening, and the interstitial inflammatory cell infiltration was lighter. The lung lesion scores of mice also suggested that both FI and IDR could significantly alleviate the pathological changes caused by COPD on lung tissue.

### 3.6 IDR and FI attenuated the development of immune cell infiltration in the lung tissue of COPD mice

**Figure 4:**
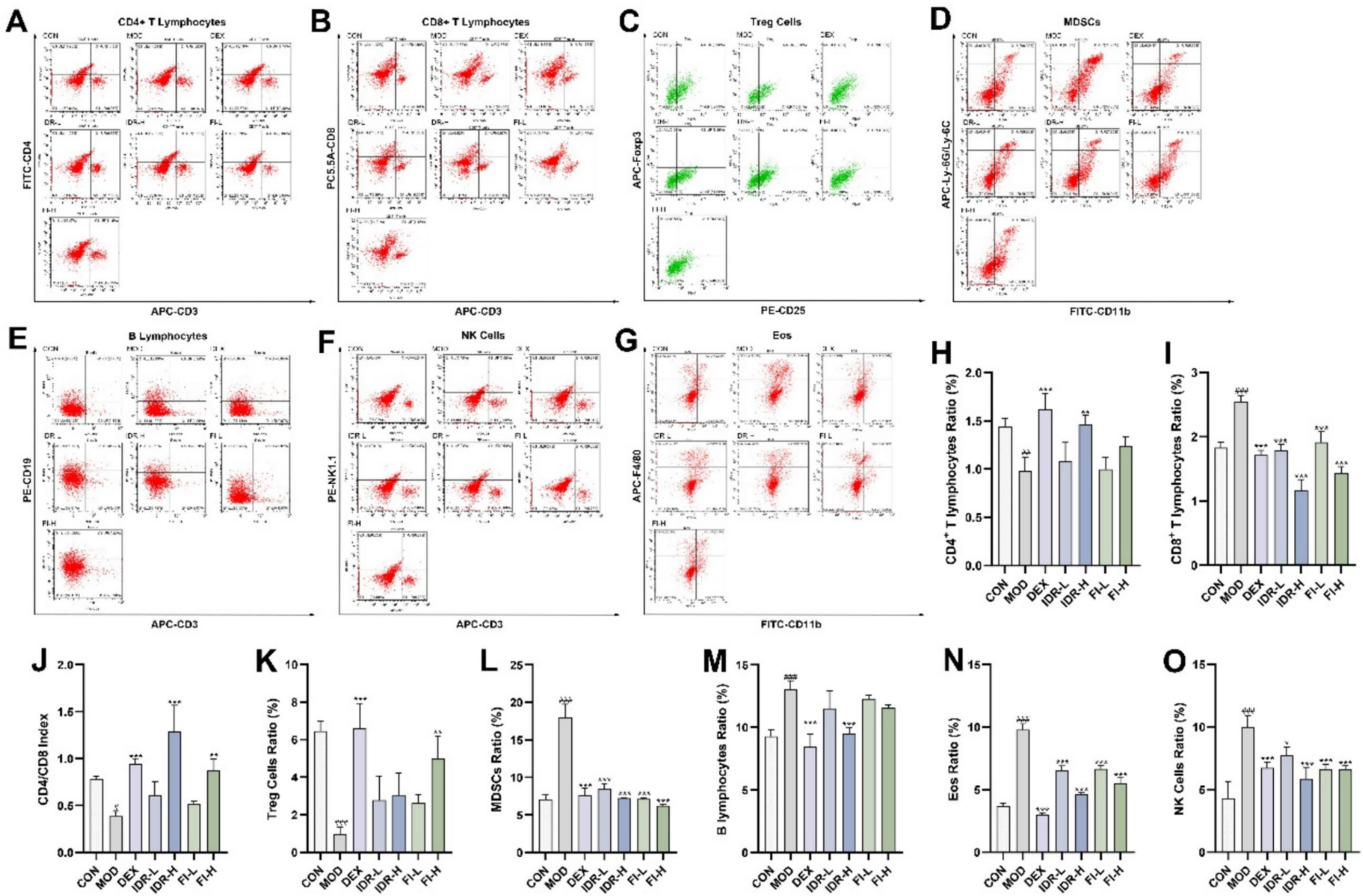
IDR and FI modulated changes in immune cell infiltration caused by COPD. Flow cytometric analysis of **(A)** CD4^+^ T lymphocytes, **(B)** CD8^+^ T lymphocytes, **(C)** Treg cells, **(D)** MDSCs, **(E)** B lymphocytes, **(F)** NK cells, and **(G)** Eos. Proportion of **(H)** CD4^+^ T lymphocytes, **(I)** CD8^+^ T lymphocytes. **(J)** CD4/CD8 index. Proportion of **(K)** Treg cells, **(L)** MDSCs, **(M)** B lymphocytes, **(N)** NK cells, and **(O)** Eos. Data were expressed as mean ± SD, ^###^*p* < 0.001 vs. CON group, ^*^p < 0.05, ^**^p < 0.01, and ^***^p < 0.001 vs. MOD group (*n* = 3).

The infiltration of immune cells in mouse lung tissue was observed using flow cytometry. The percentage of CD4^+^ T lymphocytes and Treg cells in mouse lung tissue following COPD decreased compared with the control group (*p* < 0.01, *p* < 0.001), while the percentage of CD8^+^ T lymphocytes, MDSCs, B lymphocytes, Eos and NK cells significantly increased (*p* < 0.001), and the level of CD4/CD8 decreased (*p* < 0.05) (Figure 4). Intervention with IDR and FI can significantly alleviate this change in immune cell infiltration in the lung tissue of COPD mice (*p* < 0.05).

## 4 Discussion

In this study, we compared the data obtained from the patients with COPD and normal individuals to identify relevant differential genes and their enriched BPs and signaling pathways. Our results suggested that COPD are immune-related. We analyzed the immune infiltration of the disease and selected potential Chinese medicinal herbs with therapeutic effects based on the above results. We used IDR and FI for confirmatory animal experiments and found that both treatments produced therapeutic effects in COPD mice.

Identified as a mitochondrial acyl carrier protein, NADH: ubiquinone oxidoreductase subunit AB1 (NDUFAB1) is involved in lipoic acid synthesis via the type II fatty acid biosynthesis pathway (FAS II) and also serves as an accessory subunit of complex I (7, 8). A study demonstrated that NDUFAB1 regulates mitochondrial activity and ROS metabolism via electron transport chain complexes, thereby controlling asthma development, and acts as a hub gene in asthmatic patient samples (9). Ribosomal protein S26 (RPS26), as a ribosomal protein, is involved in and regulates a variety of ribosome-related biological processes. In a recent study, RPS26 was found to be underexpressed in patients with COVID-19, and this regulation appears to be associated with immune cell surface proteins CD33 and CD48 (10). Previous studies have also shown that RPS26 is associated with SARS-CoV-2 that persists in COVID-19 patients, and RPS26 is in turn a marker gene that determines the severity of COVID-19 infection in both T and B cells (11). Leptin (LEP) is a multifunctional cytokine that exerts diverse biological effects, such as modulating immune response, promoting angiogenesis, and facilitating wound healing (12). Earlier studies have found that LEP can be used as a marker molecule based on inflammation in COPD patients (13), and the negative correlation between LEP and pulmonary fibrosis has also been demonstrated (14). Similarly, LEP receptors are expressed in various respiratory cells, including airway epithelial cells and smooth muscle cells, as well as in the lung tissue, indicating a strong association between LEP and respiratory diseases (15, 16). Phospholipase C beta 2 (PLCB2) is a phosphatidyl C family member, which is generally considered to be a gene associated with tumor immune response. In a screen for lung cancer prognostic genes, it was found to correlate with the amount of immune cell infiltration such as CD69 and M0 macrophages (17). In the meantime, the association of PLCB2 with NK cells has also been demonstrated (18). In addition, although stearoyl-CoA desaturase (SCD) (19), single DNA binding protein 1 (SSBP1) (20), fumarate hydratase (FH), TNF receptor superfamily member 9 (TNFRSF9) (21), and valyl-tRNA synthetase 1 (VARS1) (22) have not yet been found to have specific functions in respiratory-related diseases, a large number of scholars believe that these genes play an important role in the immune response of some diseases. So we suggest that their regulation of immune function may be the main path to play a role in COPD. These findings reinforce the credibility of gene chip data analysis outcomes, identifying potential therapeutic targets for COPD. Doubtfully, retina and anterior neural fold homeobox 2 (RAX2) is a gene highly associated with ocular diseases rather than with other diseases or immune function (23). However, it was also identified as a differential gene in our study, and the association of RAX2 with COPD or a broader immune response seems to need to be further explored and clarified. The enrichment analyses of differentially expressed genes using GO and KEGG databases revealed that entries closely associated with patient immunoregulatory activity were mainly enriched in BPs and signaling pathways, including B cell-mediated immunity, regulation of inflammatory responses, and defense mechanisms against Gram-negative bacteria. These insights highlight the intricate immune mechanisms involved in the onset and progression of COPD, encompassing diverse immune cells and factors.

Recent studies have demonstrated that numerous inflammatory factors contribute to immune cell infiltration and the production/release of destructive enzymes, and that COPD patients with these mediators exhibit progressive lung damage associated with airway remodeling, including central airway, distal airways, and lung parenchyma alterations (24). Furthermore, oxidative stress resulting from inhaling harmful gases and particles can provoke a substantial inflammatory reaction in the airways and lung tissue, resulting in pathological changes characteristic of COPD. Innate and adaptive immune responses form the foundation of inflammation in COPD and are central to the disease’s development, involving key immune cells such as macrophages, neutrophils, dendritic cells, and lymphocytes (25). Among these cells, macrophages are a primary source of cytokines and inflammatory mediators within the airways. Along with phagocytic granules, bacteria, and apoptotic cells, the release of inflammatory mediators also attracts other inflammatory cells, particularly neutrophils. These neutrophils travel from pulmonary capillaries to airways, where they combat pathogens through reactive oxygen radicals, antimicrobial proteins, and enzymes that break down harmful substances. Excessive production, release, and inadequate neutralization of these potentially damaging molecules have been linked to tissue damage in COPD (26). Moreover, these infiltrative inflammatory immune cells can trigger adaptive immune responses by regulating antigen-presenting cells, such as dendritic cells and macrophages, in lung tissue. The cells mediating adaptive immune response are mainly T lymphocytes and B lymphocytes. T lymphocytes are mainly divided into CD4^+^ T and CD8^+^ T cell subsets. CD4^+^ T cells can further differentiate into T helper 1 (Th1) cells and T helper 2 (Th2) cells and participate in cellular and humoral immunity, and Th1/Th2 dysregulation is associated with various chronic inflammatory airway diseases such as asthma and chronic bronchitis. Treg cells also belong to the CD4^+^ T cell subset and have immunosuppressive effects. In COPD patients, overexpression of proinflammatory cells may weaken the anti-inflammatory effect of Treg cells. Treg cell levels and expression of its factor interleukin (IL)-10 and transcription factor Foxp3 were decreased in a cigarette induced mouse COPD model, and this decrease was significant with increasing duration of cigarette smoke exposure (27, 28). CD8^+^ T cells are capable of secreting molecules that destroy infected and tumor cells. B lymphocytes can produce immunoglobulins to mediate humoral immunity. These immune responses, particularly the downstream adaptive immune response, persist in COPD and will lead to the persistence of lung tissue destruction after cessation of chronic smoking (29). MDSCs are immature heterogeneous cell populations differentiated at different stages by myeloid-derived cells that increase local oxygen free radical content and inflammatory cytokines while inhibiting T cell proliferation and immune function in inflammatory diseases, traumatic stress, as well as the tumor microenvironment (30, 31). Meanwhile, lung neoplastic lung disease mechanisms may also be associated with immunosuppression of MDSCs, such as pulmonary fibrosis, pulmonary hypertension, and chronic pneumonia (32, 33). On the other hand, the role of MDSCs in COPD disease progression remains controversial (34, 35), and the correlation between the two remains to be further explored. Eos accumulate in tissues of COPD patients and participate in the inflammatory response through the secretion of cytokines, chemokines, and degranulation. Specifically, Eos secretes profibrotic transforming growth factor-β, stimulates fibroplasia, increases collagen production, and in turn induces respiratory fibrosis and organ remodeling (36). Meanwhile, IL-13 produced by Eos was able to induce matrix metalloproteinase-12 secretion by rat alveolar macrophages, which in turn induced emphysema (37). However, the specific correlation between changes in immune cell levels caused by COPD is still unclear. The study provides some references for subsequent related studies.

Pharmacological studies have demonstrated the therapeutic effect of *Isatis indigotica* Fortune (Brassicaceae), *Platycodon grandiflorus* (*Jacq*.) A. DC. (Campanulaceae), *Panax ginseng* C.A.Mey (Araliaceae), FI, *Chrysanthemum morifolium* Ramat. (Compositae) and *Cinnamomum cassia* Presl (Lauraceae) on COPD and other respiratory diseases (38–42). “Open lung qi relieves cough and asthma, dispels spleen damp-heat, dispels liver heat to promote blood circulation, and tonifies kidney essence for full vitality.” Therefore, the analysis results of this study are in line with the syndrome differentiation treatment of COPD using TCMs.

Analysis of the immune infiltrative properties of COPD reveals the impact of COPD on lung tissue immune function. Similarly, using the potential therapeutic agent FI and its main active component IDR, both of them were found to alleviate COPD-induced lung lesions to varying degrees, and this effect may be tested by counteracting abnormal infiltration of immune cells in the body of COPD mice. Interestingly, in COPD model mice, the key features of associated immune cell infiltration were again consistent with immune infiltration analysis relying on gene chips regardless of the presence of FI and IDR intervention, which suggests further research directions and potential applications of TCMs by regulating immunotherapeutic diseases.

In contemporary TCM-related bioinformatics research, a prevalent issue stems from the restricted technical proficiency of TCM databases and analytical techniques, which can compromise the dependability of the data and results. Additionally, the sample size in our study was limited, potentially affecting the precision of our analysis outcomes to a certain extent. To enhance the reliability of future findings, research should employ larger sample sizes and conduct additional experimental replicates. Furthermore, some of the core target genes identified have not been conclusively linked to respiratory diseases and warrant further exploration to validate their association.

## 5 Conclusion

This study reports potential therapeutically active TCMs screened based on the BPs affected by COPD and immune cell infiltration reactions. Further, we found that IDR and FI improved COPD in our model mice, possibly by regulating host immune levels. Thus, from the perspective of future research, our study not only provides new evidence on the mechanism of TCMs against COPD but also provides new ideas for screening potential therapeutic Chinese medicines against COPD.

## Data Availability

The original contributions presented in the study are included in the article, further inquiries can be directed to the corresponding author.

## Footnotes

^1^ https://string-db.org/

^2^ http://www.coremine.com/
